# Global trends in sustainable healthcare research: A bibliometric analysis

**DOI:** 10.1016/j.fhj.2025.100251

**Published:** 2025-04-11

**Authors:** Ana Raquel Nunes, Jeremy Dale

**Affiliations:** Warwick Medical School, University of Warwick, Coventry CV4 7AL, United Kingdom

**Keywords:** Sustainable healthcare, Resilient healthcare, Climate change, Sustainability

## Abstract

**Introduction:**

This study maps the development of sustainable healthcare research, identifying key contributors, collaborative networks and emerging trends.

**Objective:**

To provide a comprehensive overview of the sustainable healthcare research landscape, offering insights to inform strategic planning, resource allocation and future research.

**Methods:**

A bibliometric analysis of 842 publications up to May 2024 was conducted using the Web of Science Core Collection. The analysis examined publication year, authorship, countries, journals, citations, research fields, subjects and methodologies. VOSviewer was used to visualise knowledge networks and research priorities.

**Results:**

Over 70% of publications emerged in the last 5 years, with the USA, England and Australia as leading contributors. Key trends include integrating environmental sustainability into healthcare and advancing green technologies.

**Conclusion:**

The findings highlight the rapid growth of sustainable healthcare research, offering valuable insights for policymakers, researchers and healthcare professionals seeking to enhance the resilience and sustainability of healthcare systems.

## Highlights

• Sustainable healthcare research is rapidly expanding and highly interdisciplinary.
• Original research dominates, with key areas in health services and environmental sciences.
• Western countries lead contributions; broader international participation is needed.
• Future efforts should focus on global collaboration, tech integration and inclusivity.

## Introduction

Healthcare systems globally face increasingly pressure from ageing populations, chronic diseases, rising costs, inefficiencies and waste, all threatening long-term sustainability.^1^ Organisations such as the World Health Organization (WHO), Organisation for Economic Co-operation and Development (OECD) and World Economic Forum (WEF) have recognised these challenges as critical to healthcare performance and sustainability.^2^

Climate change, widely regarded as the greatest global health threat of the 21st century, exacerbates health disparities and further strains healthcare systems, underscoring the urgent need for sustainable healthcare practices.^3^, ^4^, ^5^, ^6^

The healthcare sector contributes approximately 5% of global greenhouse gas emissions and has a significant role in climate mitigation.^7^ Sustainable healthcare integrates social, environmental and economic dimensions into healthcare service provision, ensuring long-term viability.^8^, ^9^, ^10^, ^11^, ^12^, ^13^, ^14^

Achieving sustainability requires systemic transformation, prioritising health promotion, wellbeing, and optimal clinical and environmental outcomes.^15^ Education and training on sustainability principles are crucial for fostering innovation and leadership in this field.^14^^,^^16^, ^17^, ^18^, ^19^

Despite the growing interest and investments, sustainable healthcare literature remains fragmented across disciplines, posing challenges for researchers and policymakers. Bibliometric analysis^20^, ^21^, ^22^ offers a valuable method for mapping scientific literature, identifying research trends and pinpointing gaps.

This study conducts a bibliometric analysis of publications on sustainable healthcare up to May 2024. The objectives are to map research development, highlight key contributors and collaborative networks, and uncover emerging trends and gaps. The findings provide insights for researchers, practitioners, funders and policymakers aiming to advance sustainability in healthcare systems.

## Methods

### Database

The Web of Science (WoS) Core Collection, a comprehensive academic repository^23^ was used to retrieve publications.

### Search strategy

The search included the keywords ‘sustainable healthcare’ OR ‘sustainable health care’. Terms such as ‘climate-resilient healthcare’, ‘low-carbon healthcare’ and ‘planetary health’ were excluded to maintain focus. Future research could explore these intersections in greater detail. The search applied a Topic filter (Title, Abstract, Keyword), ensuring relevance. No language limitations were imposed.

### Data extraction

Relevant details, including titles, authors, affiliations, keywords and citation data, were extracted for analysis.

### Data analysis and visualisation

Bibliometric scrutiny covered publication year, authors, country, publication type, journal, total citations and research areas. VOSviewer was used to create knowledge maps, assess keyword co-occurrence, co-authorship and reference co-citation networks^24^^,^^25^.

## Results

### Overview of sustainable healthcare research

A search on 14 May 2024 identified 842 publications on sustainable healthcare, dating back to 1992.

### Publication characteristics

#### Types of publications

Research articles are the most common type of publication, comprising 553 publications (65.7%). Following this, review articles account for 113 publications (13.4%), editorials for 73 (8.7%), and proceeding papers for 65 (7.7%). Other types, including meeting abstracts, letters, book chapters and reviews, make up the remaining 73 publications (8.7%) (Supplemental Figure 1).

#### Research areas

The top 10 research areas within sustainable healthcare from WoS, as shown in Supplemental Figure 2, highlight that Health Care Sciences Services (161 publications, 19.1%) leads the field, followed by Environmental Sciences Ecology (119 publications, 14.1%) and General Internal Medicine (102 publications, 12.1%). Other prominent areas include Public Environmental Occupational Health, Science Technology Other Topics, Engineering, Computer Science, Business Economics, Education Educational Research and Nursing.

#### Annual publication and citation trends

**Figure 1:**
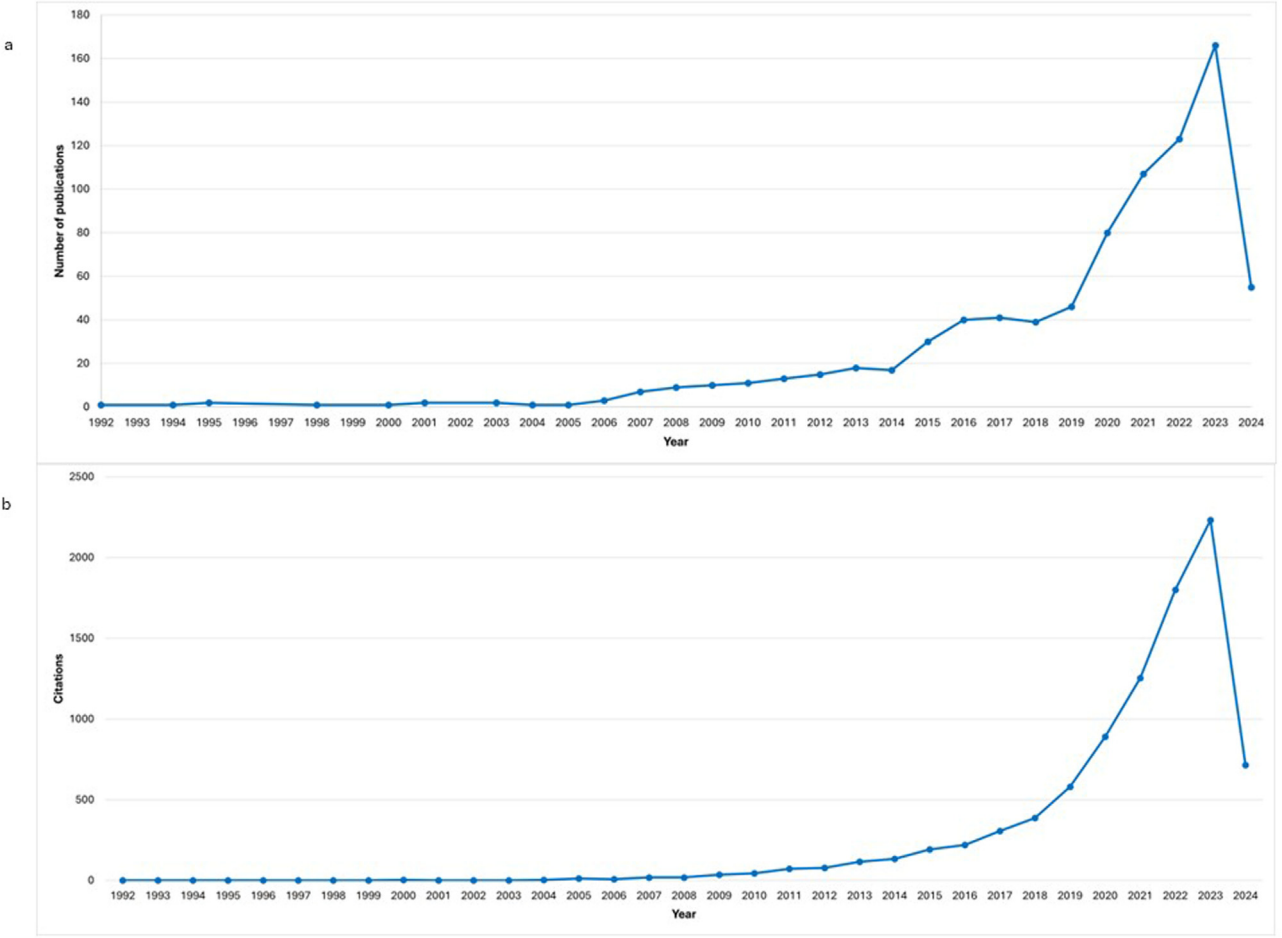
(a and b) Distribution of annual publications and citations, respectively.

The first publication in this field was published in 1992. Up until 2007, the number of publications per year did not exceed seven. However, from 2019 onwards, there has been a marked increase in the number of publications, indicating rapid development in this research area (Figure 1a). In 2023, publications in this field reached their highest citation count to date with 2,232 citations, demonstrating growing interest and recognition (Figure 1b).

#### Geographical distribution of publications

The WoS analysis identifies the USA as the leading country (ie author affiliation country) with 165 publications (19.6%), followed by England with 149 (17.7%) and Australia with 108 (12.8%). The top 10 countries also include the Netherlands, Canada, India, Italy, Germany, China and Sweden (Supplemental Table 1).

**Figure 2:**
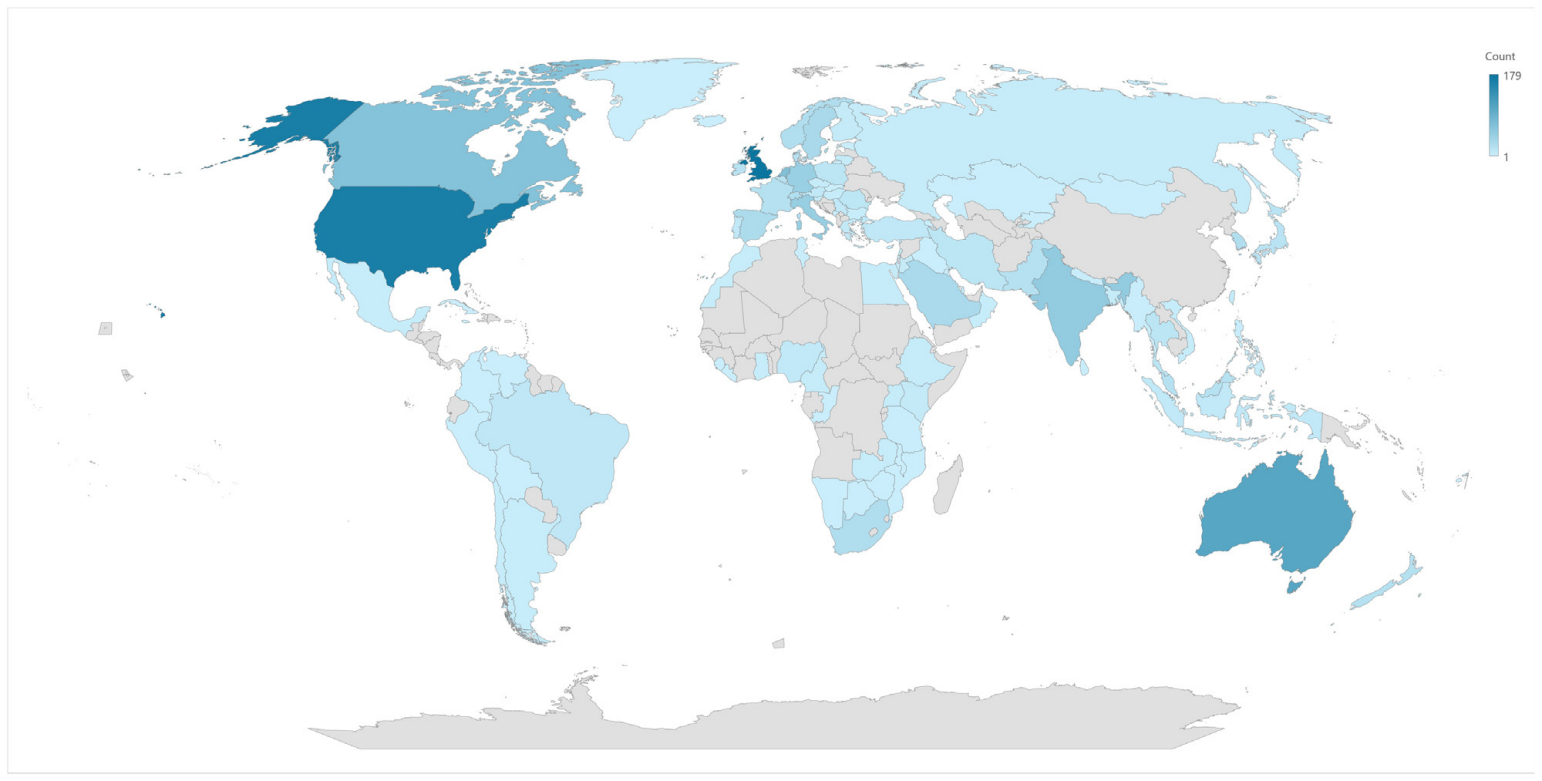
Distribution of number of publications by countries.

VOSviewer analysis, with a threshold of at least one publication and citation per country, identified 99 out of 108 countries meeting this criterion (Figure 2).

The total strength of citation links between countries (TLS) is a metric used in bibliometric analysis to measure the degree of co-authorship and citation relationships between nations. A higher TLS value indicates stronger collaborative ties between research communities. In this study, England (TLS=853), Australia (TLS=636) and the USA (TLS=605) demonstrated the strongest international collaborations (Supplemental Table 2).

**Figure 3:**
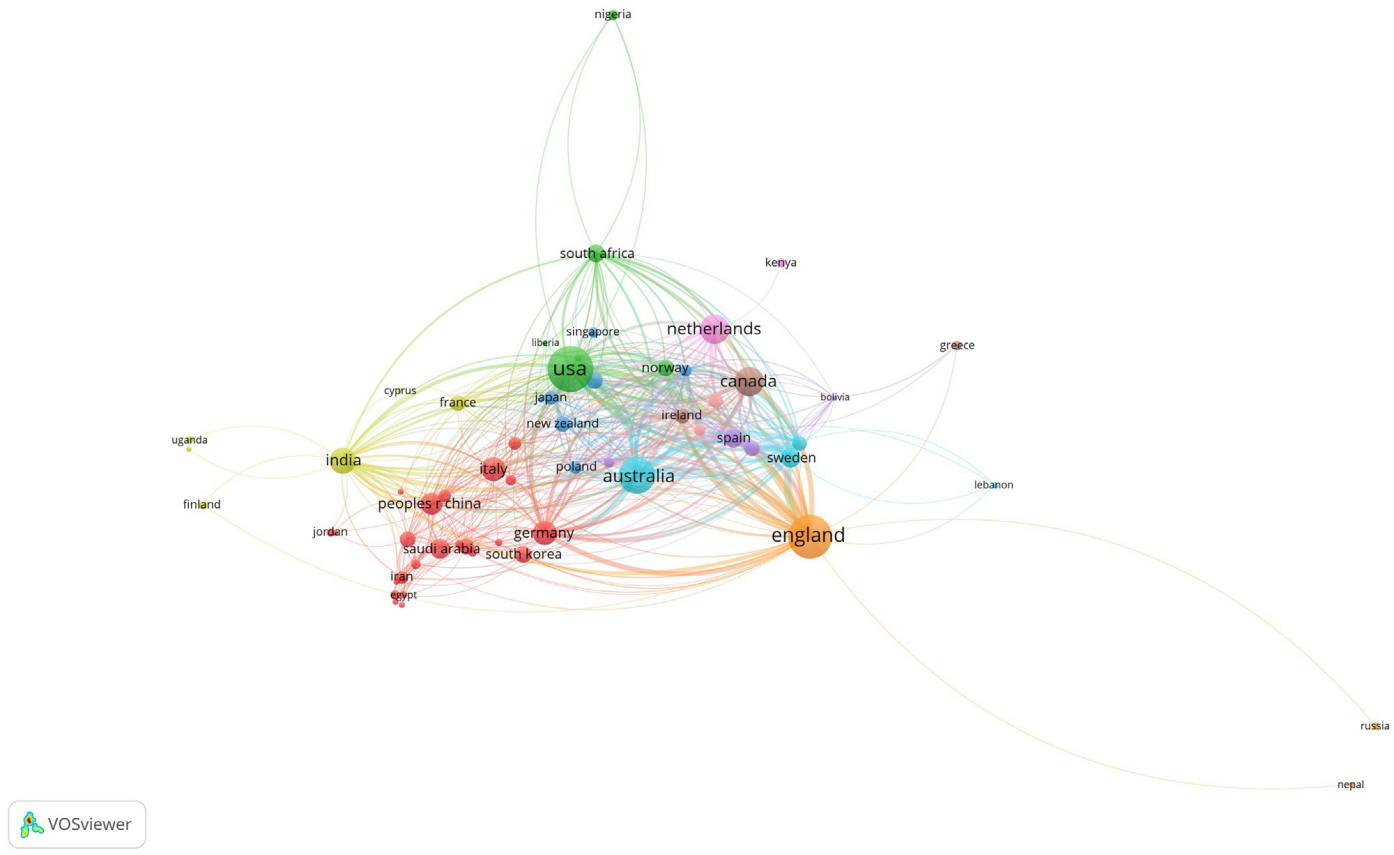
Visualisation of the most connected countries.

A network analysis shows that 62 out of the 99 connected countries form the largest set of interconnected nations, with varying strengths of co-citation links (Figure 3). The 62 countries are clustered and distinguished by different colours. Nodes indicate countries and a link between two nodes indicates that these two countries have publications co-cited. The larger the node, the more frequently the publications of this country are cited. The wider the link, the stronger the relationship between the two countries being co-cited.

#### Language distribution

English dominates as the primary language of publication, with 831 out of 842 publications (98.7%). Other languages represented include German, French, Hungarian, Italian, Japanese, Korean, Russian and Spanish, each with one to three publications (Supplemental Table 3).

#### Top cited publications

The top 10 most cited publications in sustainable healthcare span from 2004 to 2020. The most cited work, authored by Ha *et al* (2018), has 233 citations. These publications are predominantly research articles (n=7), reviews (n=2) and editorial materials (n=1). The research areas include Engineering, Biomedical, Information Systems and Environmental Sciences, reflecting interdisciplinary collaboration (Supplemental Table 4). All top 10 publications have been cited more than 100 times and all have at least two authors. Sherman and colleagues’ review (2020) has the highest number of co-authors (n=48). Additionally, half of the publications are international collaborations, which shows the level of collaboration between countries in this field of research (ie authors from institutions across countries).

### Institutional characteristics

Among the 1,706 institutions included in all publications in WoS and contributing to the body of sustainable healthcare research, 1,298 institutions met the criteria for a minimum of one publication and citation. For each of the 1,298 institutions, the total strength of the co-authorship links with other institutions was calculated and the institutions with the greatest total link strength were selected. Some of the institutions are not connected to each other, as a result the largest set of connected institutions consists of 642 (Supplemental Figure 3). The 642 institutions are clustered and distinguished by different colours. Nodes indicate institutions, and a link between two nodes indicates that these two institutions have publications co-cited. The larger the node, the more frequently the publications of this institution are cited. The wider the link, the stronger the relationship between the two institutions being co-cited.

The top 10 most influential institutions and related information are presented in Supplemental Table 5. Institutions are presented in descending order of citations. Australia, USA and England account for six of ten institutions, while the remaining are in the Netherlands, South Korea, Scotland and Canada. The University of Sydney stands out with the highest number of citations (n=265). Although both the Ulsan National Institute of Science and Technology and University of London Imperial College of Science, Technology and Medicine have only one publication each in this topic, they are far ahead of the other institutions in terms of the average number of citations. These two institutions ranked first (AC=233) and second (AC=198) in the average number of citations, and each publication was cited by an average of 233 and 198 publications, respectively. The University of Pittsburgh and Manchester Metropolitan University had three publications each in this topic and are ranked fourth (C=235) and eighth (C=204), with an average number of citations of 78.3 and 68 citations, respectively. The University of Sydney and University of Maastricht received the highest number of citations and publications, but only received an average of 20.38 and 16.13 citations, respectively. It can be seen that the average number of citations does not always correspond to the number of publications or the number of citations.

A total of 1,706 institutions have published relevant publications, of which 49 have at least five publications. For each of the 49 institutions, the total strength of the co-authorship links with other institutions was calculated and the institutions with the greatest total links strength are presented. Some of the 49 institutions are not connected to each other, the largest set of connected institutions consists of 47 institutions (Supplemental Figure 4). The size of a node indicates the total link strength (TLS) of an institution, and a link between two nodes indicates that the two institutions have a collaborative relationship. The wider the link, the higher the collaboration strength between the two institutions. The larger the node, the higher the total strength of collaboration between the institution and others.

The top 11 institutions with the strongest cooperation relationship are presented in Supplemental Table 6. The 11 institutions are presented by TLS in descending order, and four have 75% or more of their publications in collaboration with other institutions. Monash University (Australia) has the largest number of publications and largest TLS, having closest collaboration with Bond University (Australia), University of Sydney (Australia), University of Melbourne (Australia), Deakin University (Australia) and University of California (San Franscisco). As the institution with the second largest number of publications, Maastricht University (the Netherlands) has closest collaborations with Erasmus University (the Netherlands) (Supplemental Figure 4).

### Author characteristics

Of the 3,282 authors identified, 2,453 met the threshold for at least one publication and citation. For each of the 2,453 authors, the total strength of co-authorship links with other authors was calculated. The authors with the greatest total link strength were selected. Some of the authors are not connected to each other, and the largest set of connected authors consists of 76 (Supplemental Figure 5). The 76 authors are clustered by different colours. The size of a node indicates the number of citations of an author. A connection between two nodes indicates that the two authors have co-cited publications.

The top 10 most influential authors are presented in Supplemental Table 7 and ranked in descending order of citations. Melissa M Bilec is the most cited author with significant contributions in the field (C=235), but ranks in the bottom three in the average of citations (AC=78.33). Other leading authors include Alastair J Moss, David E Newby, Edward D Nicol and Michelle C Williams, who co-authored one publication and all rank fifth in the number of citations (C=197) and second in the average citations (AC=197), as their publication has been cited 197 times.

### Publication and citation sources

Elsevier, with 128 publications, is the leading publisher in this field, followed by Springer Nature (P=114) and MDPI (P=93) (Supplemental Table 8).

The classification of publications by journals as per Supplemental Table 9 revealed that *Sustainability* (P=52) and *Medical Teacher* (P=26) dominated the top two rankings, followed by the *British Medical Journal* (P=13). The dominance of *Sustainability* as a top journal (52 publications) may partly be attributed to its broad scope and high publication volume.

**Figure 4:**
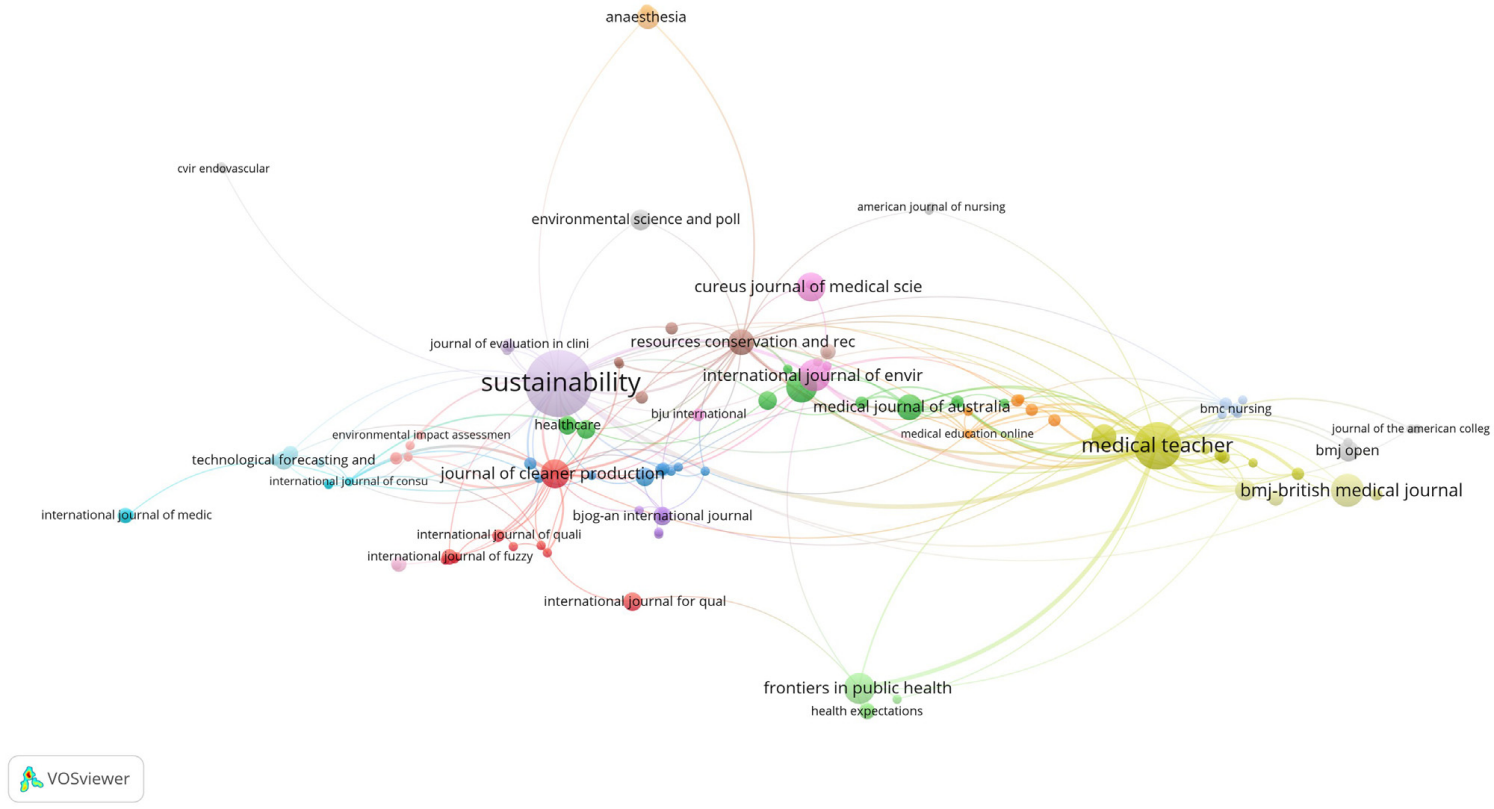
Visualisation of top journals.

Of the 524 sources, 398 met the threshold of at least one publication and citation. For each of the 398 sources, the total strength of the citation links with other sources was calculated. The sources with the greatest total link strength were selected (Figure 4). Some of the 398 sources are not connected to each other, and the largest set of connected sources consists of 113 sources.

### Keyword analysis

**Figure 5:**
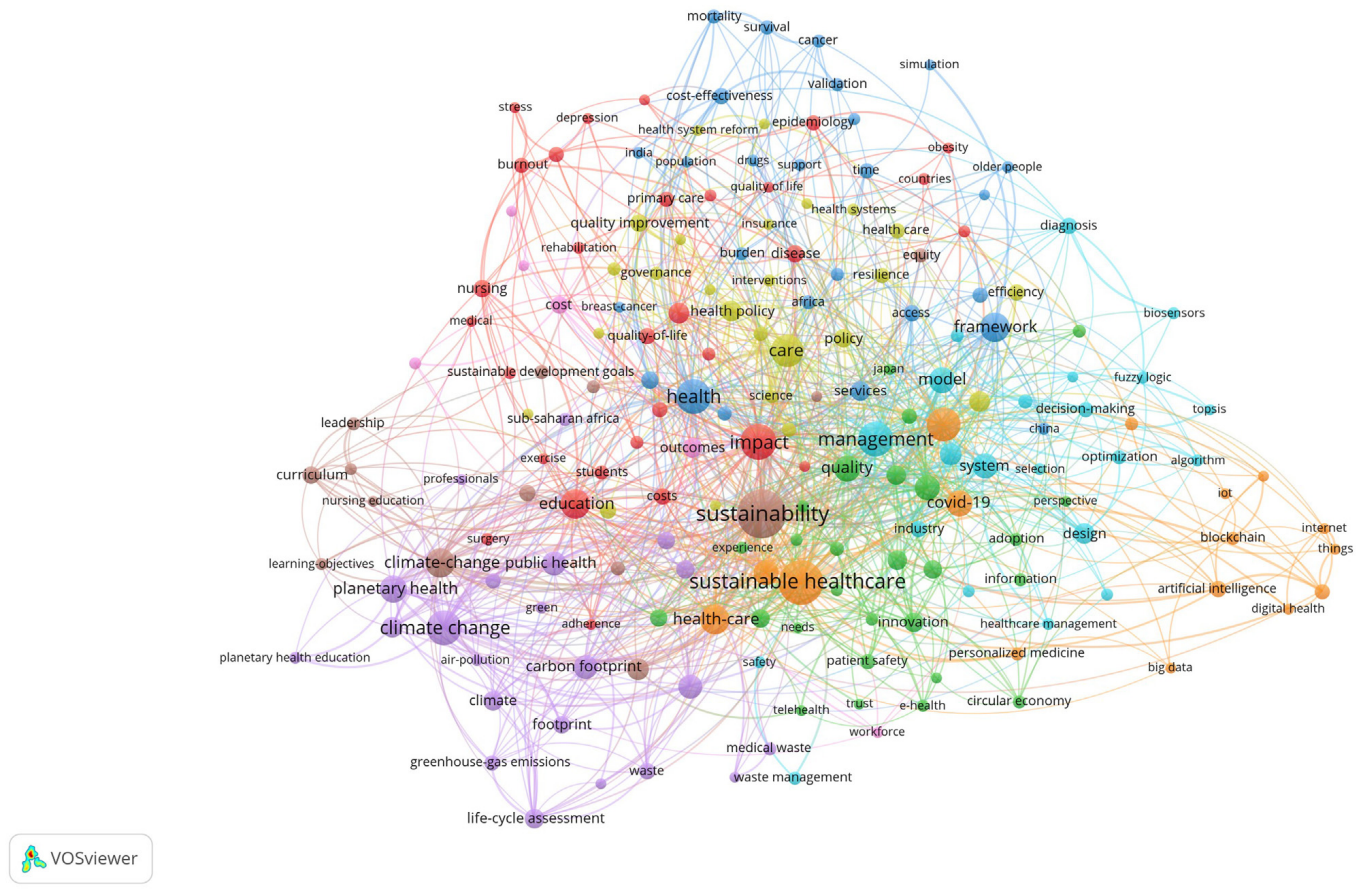
Visualisation of co-occurrence network of the most frequent keywords.

Of the 3,715 keywords, 188 met the threshold of at least five occurrences. For each of the 188 keywords, the total strength of the co-occurrence links with other keywords was calculated. The number of keywords with the greatest total link were selected. The network visualisation (Figure 5) shows dense interconnections among these keywords, reflecting the multidisciplinary nature of sustainable healthcare research. Nodes represent keywords, and a link between two nodes indicates that the two keywords appear in the same publication. The larger the node, the more frequently the keyword appears. The thicker the link, the higher the number of co-occurrences.

The 188 keywords were categorised into nine clusters with different colours based on their thematic similarities (Supplemental Table 10).

Emerging topics in sustainable healthcare research are reflected in the keyword analysis and thematic clustering of publications (Figure 5). The most frequently occurring keywords, including ‘sustainability’ (95), ‘sustainable healthcare’ (78) and ‘impact’ (52), highlight the field’s central focus on assessing and implementing sustainability in healthcare systems (Supplemental Table 11). The prominence of ‘management’ (49) and ‘framework’ (34) suggests a growing interest in strategic approaches to sustainability. Environmental concerns are evident in the frequence of ‘climate change’ (48) and ‘climate-change’ (35), indicating a strong research focus on healthcare’s role in mitigating impact and adapting to climate challenges. The inclusion of ‘education’ (34) underscores the increasing emphasis on integrating sustainability principles into medical and healthcare training to equip health professionals with environmental knowledge. Operational aspects of sustainability are also reflected in keywords such as ‘health’ (48), ‘healthcare’ (46), ‘care’ (43) and ‘health-care’ (34), highlighting that studies are examining the implementation of sustainable practices into clinical practices, resource management, and patient care models and pathways.

## Discussion

Sustainable healthcare research is growing rapidly, with strong international collaboration. Most publications are empirical research articles (65.7%), with the USA, England and Australia as leading contributors. Over 70% of studies were published in the past 5 years. Research themes span environmental sustainability, technological innovations and policy development.

Findings confirm the interdisciplinary nature of the field^11^^,^^26^ and highlight the prominence of original research articles, reflecting an ongoing focus on empirical evidence for practice and policy.^4^^,^^11^^,^^15^ Identified keyword clusters correspond with themes in prior literature, including education,^16^^,^^27^ patient-centred care,^3^^,^^4^ technological advancements^28^ and policymaking.^1^^,^^29^^,^^30^ These clusters reflect the multifaceted nature of sustainable healthcare.

The geographical distribution of research outputs aligns with previous studies, showing a predominance of research from Western countries such as the USA, England and Australia.^1^ However, this study emphasises the need for broader international participation to enhance the global relevance of sustainable healthcare solutions, echoing calls for increased global collaboration.^2^^,^^5^^,^^31^, ^32^, ^33^

This study also notes an increase in publications and citations in recent years, indicating growing interest and investment in sustainable healthcare research. This trend highlights the importance of sustainability in healthcare and the need for continued support to drive innovation in the field.^2^^,^^16^

One significant limitation of bibliometric analysis is its bias towards research from English-speaking countries, which can distort global research trends and underrepresent contributions from non-English-speaking regions. This can lead to an overemphasis on findings from a few high-income countries, affecting global research agendas and policy decisions. To ensure a comprehensive understanding and more equitable decision-making, it is crucial to identify and address these biases, highlighting underrepresented research from diverse linguistic and regional contexts.^34^

This study effectively used bibliometric methods to analyse trends in sustainable healthcare research, providing valuable quantitative insights, mapping scientific data, and identifying research priorities. Strengths include a comprehensive dataset, robust methodology and visualisation techniques. However, limitations include the exclusion of non-WoS-indexed studies and qualitative insights. Terms such as ‘climate-resilient healthcare’, ‘low-carbon healthcare, and ‘planetary health’ could also fall under the sustainability domain; however, this study prioritised a targeted approach to ensure clarity and depth in bibliometric mapping. Future studies could explore the intersections between these fields in greater detail.

As part of a bibliometric study, it is not possible to systematically categorise publications by medical specialty, as bibliometric analysis relies on metadata rather than full-text content. While understanding the engagement of specific medical specialties in sustainability is valuable, achieving this requires a scoping or systematic review.

Additionally, there is a risk of language bias, particularly in non-English publications, which could result in the exclusion of relevant research. Addressing these limitations would require broader inclusion of non-English terms and publications.

Findings highlight key trends in sustainable healthcare research, emphasising the need for interdisciplinary approaches that integrate health services, environmental sustainability and clinical practice. The significant rise in publications and citations since 2019 reflects growing interest and investment in the field. However, the dominance of research from Western countries indicates a need for broader international participation and multilingual publications to enhance global relevance. Strong international collaborations are crucial for advancing the field, particularly in leveraging technology to address complex healthcare challenges. Findings highlight the need for interdisciplinary collaboration and increased global participation, and the integration of technological innovations to build resilient and sustainable healthcare systems.

The study advocates for fostering global collaboration, especially involving developing countries, to create adaptable solutions for diverse contexts. Expanding research to applied studies can bridge the gap between theory and practice, influencing policy and healthcare. Investment in technological advancements, such as digital health, AI and the Internet of Things (IoT), is crucial for improving healthcare delivery and reducing environmental impact. Promoting interdisciplinary research, enhancing education, and increasing visibility through open access and international conferences are recommended to drive innovation. Building networks among academia, industry and government will support sustainable practices, while addressing ethical and equity concerns is essential to ensure inclusive and equitable healthcare systems for all populations, particularly vulnerable communities.

Future research should explore intersections between sustainable healthcare, climate resilience and planetary health. Expanding analysis to non-English publications can enhance global representation.

## Conclusion

This bibliometric analysis provides a comprehensive overview of sustainable healthcare research landscape, highlighting its rapid growth, key contributors and emerging trends. Addressing identified gaps, particularly regional disparities and medical specialty engagement, will be crucial for advancing the field. The findings serve as a resource for researchers, practitioners and policymakers dedicated to enhancing healthcare sustainability.

## Patient and public involvement

Patients and/or the public were not involved in the design, conduct, reporting or dissemination plans of this research.

## Ethics approval and consent to participate

Not applicable.

## Data availability

All data is publicly available.

## CRediT authorship contribution statement

**Ana Raquel Nunes:** Writing – review & editing, Writing – original draft, Visualization, Methodology, Investigation, Formal analysis, Data curation, Conceptualization. **Jeremy Dale:** Writing – review & editing.

## Declaration of competing interest

The authors declare that they have no known competing financial interests or personal relationships that could have appeared to influence the work reported in this paper.

## Appendix Supplementary materials

Image, application 1

Image, application 2

Image, application 3

Image, application 4

Image, application 5

Image, application 6

Image, application 7

Image, application 8

Image, application 9

Image, application 10

Image, application 11

Image, application 12

Image, application 13

Image, application 14

Image, application 15

Image, application 16
