# Supplementary material for: Global trends in sustainable healthcare research: A bibliometric analysis
Source: Future Healthc J. 2025 Apr 11;12(2):100251. doi: 10.1016/j.fhj.2025.100251 (PMC12133695; doi:10.1016/j.fhj.2025.100251)
Supplement: Supplementary file 1 [file mmc1.docx]

**Online Supplemental Table 1.** Top 10 most productive countries

| Rank | Country | P | % (N=842) |
| --- | --- | --- | --- |
| 1 | USA | 165 | 19.6% |
| 2 | England | 149 | 17.7% |
| 3 | Australia | 108 | 12.8% |
| 4 | Netherlands | 67 | 8.0% |
| 5 | Canada | 65 | 7.7% |
| 6 | India | 52 | 6.2% |
| 7 | Italy | 47 | 5.6% |
| 8 | Germany | 46 | 5.5% |
| 9 | China | 39 | 4.6% |
| 10 | Sweden | 32 | 3.8% |

*P: number of publications
