# Supplementary material for: Global trends in sustainable healthcare research: A bibliometric analysis
Source: Future Healthc J. 2025 Apr 11;12(2):100251. doi: 10.1016/j.fhj.2025.100251 (PMC12133695; doi:10.1016/j.fhj.2025.100251)
Supplement: Supplementary file 2 [file mmc2.docx]

**Online Supplemental Table 2.** Top 10 countries with the greatest total link strength

| Rank | Country | P | C | TLS |
| --- | --- | --- | --- | --- |
| 1 | England | 149 | 2484 | 853 |
| 2 | Australia | 107 | 1455 | 636 |
| 3 | USA | 165 | 2636 | 605 |
| 4 | Canada | 65 | 848 | 383 |
| 5 | Germany | 46 | 686 | 358 |
| 6 | Sweden | 31 | 398 | 262 |
| 7 | Wales | 11 | 319 | 244 |
| 8 | India | 52 | 585 | 232 |
| 9 | Ireland | 17 | 453 | 224 |
| 10 | Spain | 28 | 405 | 199 |

*P: number of publications; C: number of citations; TLS: total link strength
