# Supplementary material for: Global trends in sustainable healthcare research: A bibliometric analysis
Source: Future Healthc J. 2025 Apr 11;12(2):100251. doi: 10.1016/j.fhj.2025.100251 (PMC12133695; doi:10.1016/j.fhj.2025.100251)
Supplement: Supplementary file 3 [file mmc3.docx]

**Online Supplemental Table 3.** Languages used in publications

| Rank | Language | P | % (N=842) |
| --- | --- | --- | --- |
| 1 | English | 831 | 98.7% |
| 2 | German | 3 | 0.4% |
| 3 | French | 2 | 0.2% |
| 4 | Hungarian | 1 | 0.1% |
| 5 | Italian | 1 | 0.1% |
| 6 | Japanese | 1 | 0.1% |
| 7 | Korean | 1 | 0.1% |
| 8 | Russian | 1 | 0.1% |
| 9 | Spanish | 1 | 0.1% |

*P: number of publications
