# Supplementary material for: Global trends in sustainable healthcare research: A bibliometric analysis
Source: Future Healthc J. 2025 Apr 11;12(2):100251. doi: 10.1016/j.fhj.2025.100251 (PMC12133695; doi:10.1016/j.fhj.2025.100251)
Supplement: Supplementary file 4 [file mmc4.docx]

**Online Supplemental Table 4.** Top 10 most cited publications

| Rank | Authors | Title | C | Type | Research area | AN | IN | CN |
| --- | --- | --- | --- | --- | --- | --- | --- | --- |
| 1 | Ha et al. (2018) | Wearable and flexible sensors for user-interactive health-monitoring devices | 233 | Review | Materials Science, Biomaterials | 3 | 1 | 1 |
| 2 | Andreu-Perez et al. (2015) | From Wearable Sensors to Smart Implants-Toward Pervasive and Personalized Healthcare | 198 | Article | Engineering, Biomedical | 4 | 2 | 1 |
| 3 | Moss et al. (2017) | The Updated NICE Guidelines: Cardiac CTas the First-Line Test for Coronary Artery Disease | 196 | Article | Radiology, Nuclear Medicine & Medical Imaging | 4 | 2 | 1 |
| 4 | Curtis et al. (2017) | Translating research findings to clinical nursing practice | 151 | Article | Nursing | 4 | 9 | 1 |
| 5 | Abou-Nassar et al. (2020) | DITrust Chain: Towards Blockchain-Based Trust Models for Sustainable Healthcare IoT Systems | 141 | Article | Computer Science, Information Systems; Engineering, Electrical & Electronic; Telecommunications | 6 | 7 | 6 |
| 6 | Morhason-Bello et al. (2013) | Challenges and opportunities in cancer control in Africa: a perspective from the African Organisation for Research and Training in Cancer | 133 | Article | Oncology | 7 | 7 | 3 |
| 7 | Simmons-Mackie et al. (2007) | Communicative access and decision making for people with aphasia: Implementing sustainable healthcare systems change | 125 | Article | Audiology & Speech-Language Pathology; Linguistics; Clinical Neurology; Rehabilitation | 6 | 5 | 2 |
| 8 | Ferguson and Frydman (2004) | The first generation of e-patients - These new medical colleagues could provide sustainable healthcare solutions | 124 | Editorial Material | Medicine, General & Internal | 2 | 2 | 1 |
| 9 | Shaw et al (2021) | AMEE Consensus Statement: Planetary health and education for sustainable healthcare | 114 | Article | Education, Scientific Disciplines; Health Care Sciences & Services | 34 | 32 | 13 |
| 10 | Sherman et al. (2020) | The Green Print: Advancement of Environmental Sustainability in Healthcare | 109 | Review | Engineering, Environmental; Environmental Sciences | 48 | 66 | 8 |

*C: the number of citations; AN: the number of authors, IN: the number of institutions; CN: the number of countries
