# Supplementary material for: Global trends in sustainable healthcare research: A bibliometric analysis
Source: Future Healthc J. 2025 Apr 11;12(2):100251. doi: 10.1016/j.fhj.2025.100251 (PMC12133695; doi:10.1016/j.fhj.2025.100251)
Supplement: Supplementary file 5 [file mmc5.docx]

**Online Supplemental Table 5.** Top 10 most influential institutions

| Rank | Institution | Country | C | P | AC | TLS |
| --- | --- | --- | --- | --- | --- | --- |
| 1 | University of Sydney | Australia | 265 | 13 | 20.38 | 25 |
| 2 | Maastricht University | The Netherlands | 258 | 16 | 16.13 | 11 |
| 3 | University of Technology, Sydney | Australia | 240 | 4 | 60 | 3 |
| 4 | University of Pittsburgh | USA | 235 | 3 | 78.33 | 64 |
| 5 | Ulsan National Institute of Science & Technology | South Korea | 233 | 1 | 233 | 0 |
| 6 | University of Edinburgh | Scotland | 230 | 5 | 46 | 15 |
| 7 | University of California, San Franscisco | USA | 216 | 10 | 21.6 | 227 |
| 8 | Manchester Metropolitan University | England | 204 | 3 | 68 | 9 |
| 9 | University of Toronto | Canada | 199 | 11 | 18.09 | 56 |
| 10 | University of London Imperial College of Science, Technology and Medicine | England | 198 | 1 | 198 | 0 |

*P: number of publications; C: number of citations; AC: average citations; TLS: total link strength
