# Supplementary material for: Global trends in sustainable healthcare research: A bibliometric analysis
Source: Future Healthc J. 2025 Apr 11;12(2):100251. doi: 10.1016/j.fhj.2025.100251 (PMC12133695; doi:10.1016/j.fhj.2025.100251)
Supplement: Supplementary file 6 [file mmc6.docx]

**Online Supplemental Table 6.** Top 11 institutions with the strongest cooperation relationship

| Rank | Institution | Country | P | TLS | Total cooperation strength |
| --- | --- | --- | --- | --- | --- |
| 1 | Monash University | Australia | 18 | 16 | 88.89% |
| 2 | University of Sydney | Australia | 13 | 9 | 69.23% |
| 3 | Maastricht University | The Netherlands | 16 | 8 | 50% |
| 4 | Bond University | Australia | 13 | 7 | 53.85% |
| 5 | Deakin University | Australia | 10 | 7 | 70% |
| 6 | Centre for Sustainable Healthcare | England | 12 | 6 | 50% |
| 7 | University of California, San Franscisco | USA | 10 | 6 | 60% |
| 8 | University Notre Dame Australia | Australia | 6 | 6 | 100% |
| 9 | University of Oxford | England | 10 | 6 | 60% |
| 10 | University of Melbourne | Australia | 12 | 6 | 50% |
| 11 | University of Newcastle | England | 8 | 6 | 75% |

*P: number of publications; TLS: total link strength; Total cooperation strength=TLS/P
