# Supplementary material for: Global trends in sustainable healthcare research: A bibliometric analysis
Source: Future Healthc J. 2025 Apr 11;12(2):100251. doi: 10.1016/j.fhj.2025.100251 (PMC12133695; doi:10.1016/j.fhj.2025.100251)
Supplement: Supplementary file 7 [file mmc7.docx]

**Online Supplemental Table 7.** Top 10 most influential authors

| Rank | Author | Country | C | P | AC | TLS |
| --- | --- | --- | --- | --- | --- | --- |
| 1 | Melissa M. Bilec | USA | 235 | 3 | 78.33 | 3 |
| 2 | Cassandra L. Thiel | USA | 218 | 3 | 72.67 | 2 |
| 3 | Noe C. Woods | USA | 212 | 2 | 106 | 2 |
| 4 | Guang-Zhong Yang | England | 198 | 1 | 198 | 0 |
| 5 | Alastair J. Moss | Scotland | 197 | 1 | 197 | 1 |
| 6 | David E. Newby | Scotland | 197 | 1 | 197 | 1 |
| 7 | Edward D. Nicol | Scotland | 197 | 1 | 197 | 1 |
| 8 | Michelle C. Williams | Scotland | 197 | 1 | 197 | 1 |
| 9 | Julie Considine | Australia | 162 | 2 | 81 | 1 |
| 10 | Amy E. Landis | USA | 161 | 3 | 53.67 | 3 |

*P: number of publications; C: number of citations; AC: average citations; TLS: total link strength
