# Supplementary material for: Global trends in sustainable healthcare research: A bibliometric analysis
Source: Future Healthc J. 2025 Apr 11;12(2):100251. doi: 10.1016/j.fhj.2025.100251 (PMC12133695; doi:10.1016/j.fhj.2025.100251)
Supplement: Supplementary file 8 [file mmc8.docx]

**Online Supplemental Table 8.** Top 5 sources of publications

| Rank | Publisher | P | % (N=842) |
| --- | --- | --- | --- |
| 1 | Elsevier | 128 | 15.2% |
| 2 | Springer Nature | 114 | 13.5% |
| 3 | MDPI | 93 | 11.0% |
| 4 | Wiley | 71 | 8.4% |
| 5 | Taylor & Francis | 69 | 8.2% |

*P: number of publications.
