# Supplementary material for: Global trends in sustainable healthcare research: A bibliometric analysis
Source: Future Healthc J. 2025 Apr 11;12(2):100251. doi: 10.1016/j.fhj.2025.100251 (PMC12133695; doi:10.1016/j.fhj.2025.100251)
Supplement: Supplementary file 9 [file mmc9.docx]

**Online Supplemental Table 9.** Top 11 most active journals

| Rank | Journal | P | C | TLS |
| --- | --- | --- | --- | --- |
| 1 | Sustainability | 52 | 488 | 72 |
| 2 | Medical Teacher | 26 | 418 | 140 |
| 3 | British Medical Journal | 13 | 156 | 5 |
| 4 | International Journal of Environmental Research and Public Health | 12 | 152 | 32 |
| 5 | BMC Health Services Research | 11 | 112 | 5 |
| 6 | Frontiers in Public Health | 11 | 55 | 23 |
| 7 | Journal of Cleaner Production | 10 | 380 | 39 |
| 8 | CUREUS Journal of Medical Science | 10 | 20 | 3 |
| 9 | Resources, Conservation and Recycling | 8 | 385 | 38 |
| 10 | Medical Journal of Australia | 8 | 69 | 14 |
| 11 | PLOS ONE | 8 | 39 | 1 |

*P: number of publications; C: number of citations; TLS: total link strength
