# Supplementary material for: Global trends in sustainable healthcare research: A bibliometric analysis
Source: Future Healthc J. 2025 Apr 11;12(2):100251. doi: 10.1016/j.fhj.2025.100251 (PMC12133695; doi:10.1016/j.fhj.2025.100251)
Supplement: Supplementary file 10 [file mmc10.docx]

Supplemental Table 10

| *Cluster 1* (red nodes) focuses on ‘education’, ‘participation’, ‘primary care’, ‘recommendations’, ‘impact’, and ‘satisfaction’, indicating a focus on patient-centred care and healthcare delivery.  *Cluster 2* (green nodes) centres around keywords such as ‘circular economy’, ‘implementation’, ‘innovation’, ‘knowledge’, and ‘patient safety’, suggesting a focus on sustainability practices, technological advancements, and quality improvement.  *Cluster 3* (dark blue nodes) revolves around ‘collaboration’, ‘cost-effectiveness’, ‘framework’, ‘patient satisfaction’, and ‘simulation’, indicating a focus on healthcare systems, efficiency, and effectiveness.  *Cluster 4* (yellow nodes) encompasses ‘governance’, ‘health policy’, ‘health system reform’, ‘quality improvement’, ‘interventions’, and ‘resilience’, suggesting a focus on policymaking, system restructuring, and quality assurance.  *Cluster 5* (purple nodes) focuses on environmental aspects such as ‘carbon footprint’, ‘climate change’, ‘global health’, ‘public health’, ‘sustainable development’, indicating a focus on environmental sustainability and global health challenges.  *Cluster 6* (light blue nodes) centres around methodologies such as ‘analytic hierarchy process’, ‘decision-making’, ‘health technology assessment’, ‘management’, and ‘optimisation’, suggesting a focus on research methodologies and healthcare management strategies.  *Cluster 7* (orange nodes) focuses on technological advancements such as ‘artificial intelligence’, ‘big data’, ‘blockchain’, ‘deep learning’, and ‘digital health’, indicating a focus on innovative technologies in healthcare delivery.  *Cluster 8* (brown nodes) centres around ‘attitudes’, ‘curriculum’, ‘equity’, ‘ethics’, ‘leadership’, and ‘multiprofessional’, suggesting a focus on human factors, ethics, and leadership in healthcare.  *Cluster 9* (pink nodes) revolves around topics such as ‘cost’, ‘developing countries’, ‘emissions’, ‘outcomes’, ‘workforce’, and ‘environmental health’, indicating a focus on economic factors, global health disparities, and workforce sustainability. |
| --- |
