# Supplementary material for: Global trends in sustainable healthcare research: A bibliometric analysis
Source: Future Healthc J. 2025 Apr 11;12(2):100251. doi: 10.1016/j.fhj.2025.100251 (PMC12133695; doi:10.1016/j.fhj.2025.100251)
Supplement: Supplementary file 11 [file mmc11.docx]

**Supplemental Table 11.** Top 12 keywords with the largest occurrences

| Rank | Keyword | Occurrences |
| --- | --- | --- |
| 1 | Sustainability | 95 |
| 2 | Sustainable healthcare | 78 |
| 3 | Impact | 52 |
| 4 | Management | 49 |
| 5 | Climate change | 48 |
| 6 | Health | 48 |
| 7 | Healthcare | 46 |
| 8 | Care | 43 |
| 9 | Climate-change | 35 |
| 10 | Education | 34 |
| 11 | Health-care | 34 |
| 12 | Framework | 34 |
