# Supplementary figures and images for: Global trends in sustainable healthcare research: A bibliometric analysis
Source: Future Healthc J. 2025 Apr 11;12(2):100251. doi: 10.1016/j.fhj.2025.100251 (PMC12133695; doi:10.1016/j.fhj.2025.100251)

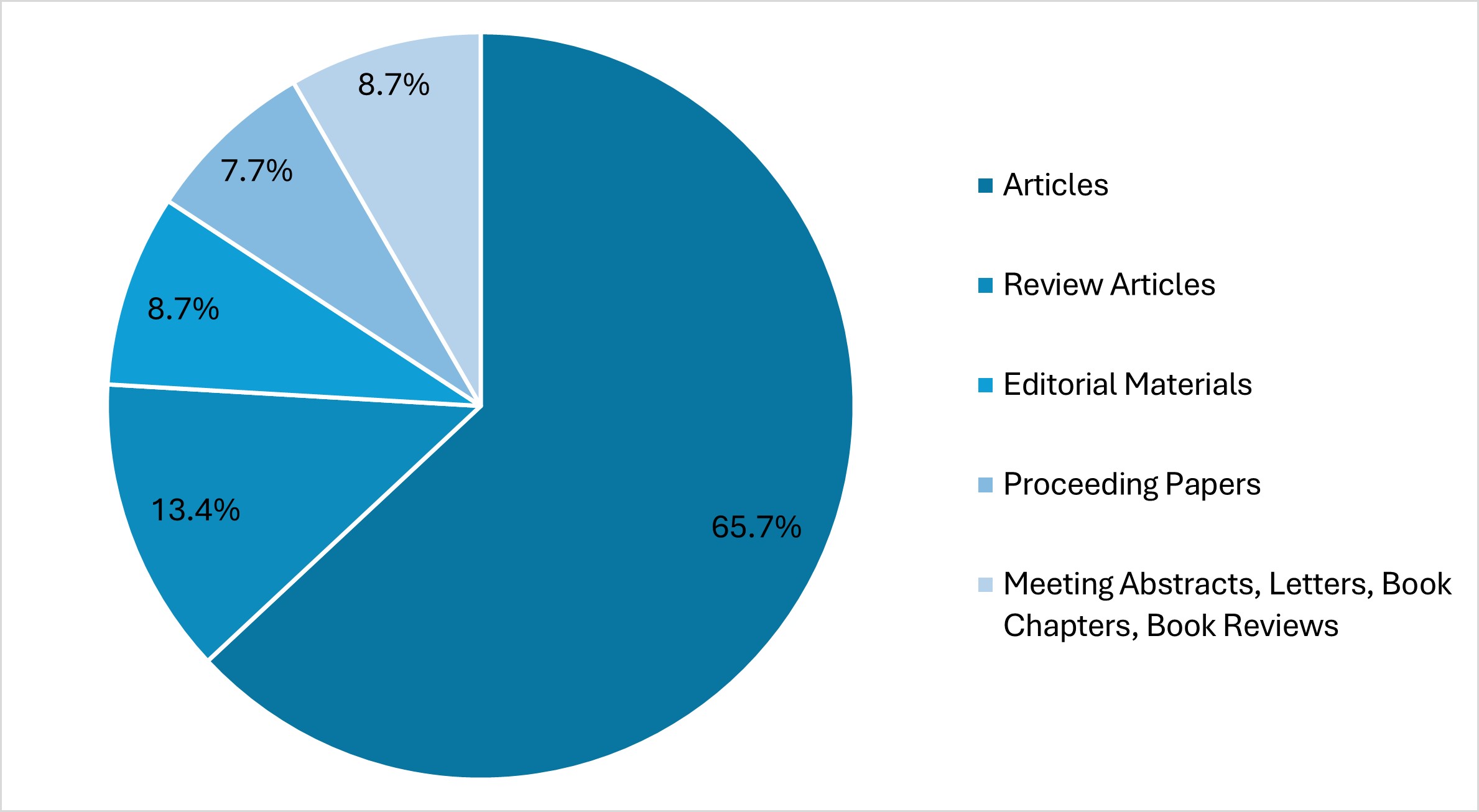

Supplement: Supplementary file 12 [file mmc12.zip › Supplemental Figure 1.jpeg]

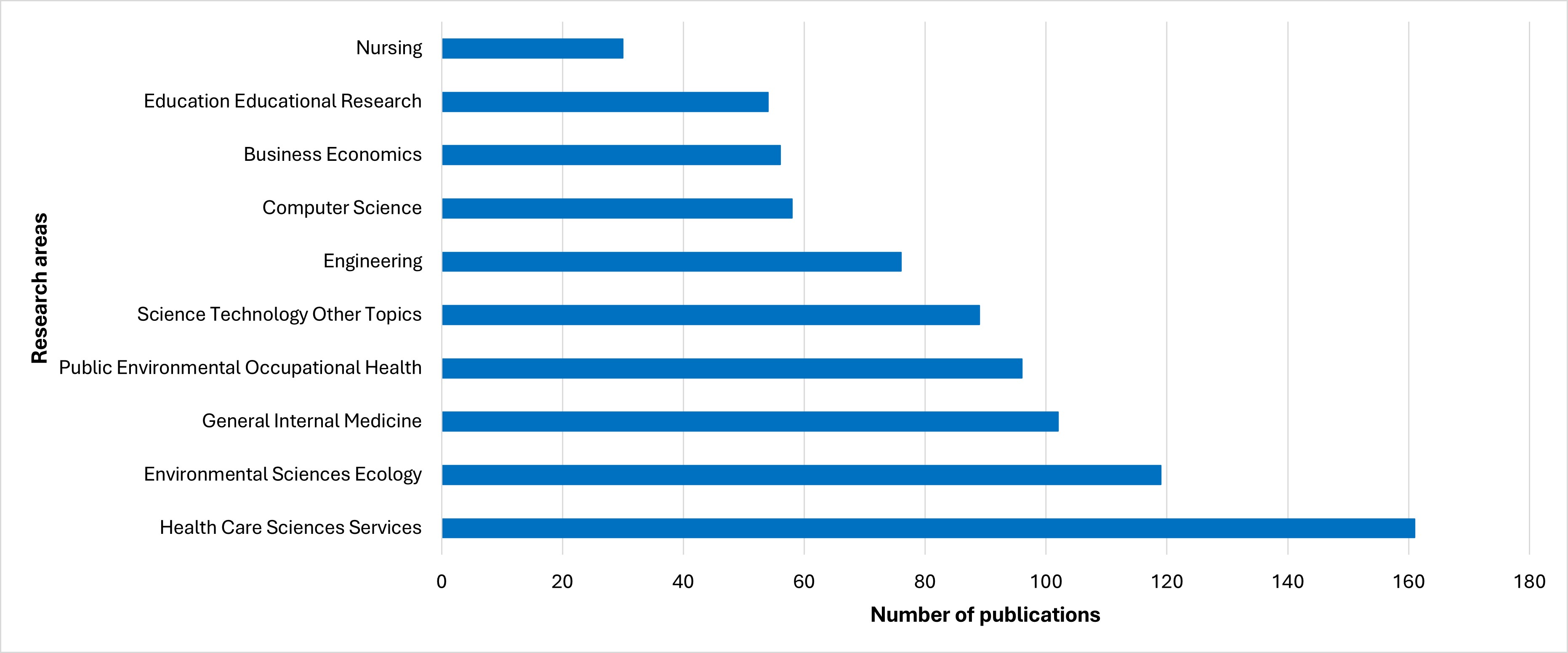

Supplement: Supplementary file 13 [file mmc13.zip › Supplemental Figure 2.jpeg]

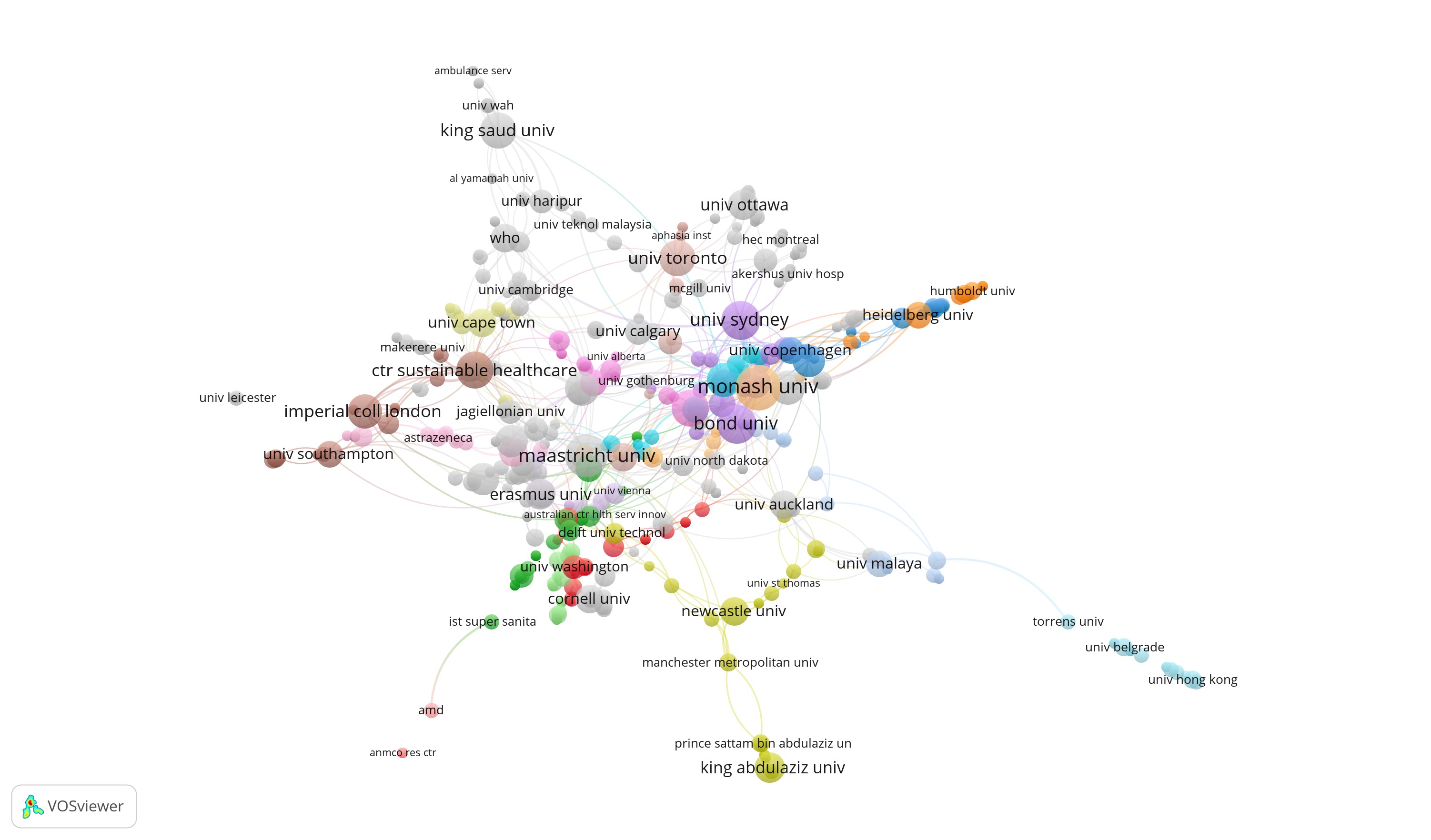

Supplement: Supplementary file 14 [file mmc14.zip › Supplemental Figure 3.jpeg]

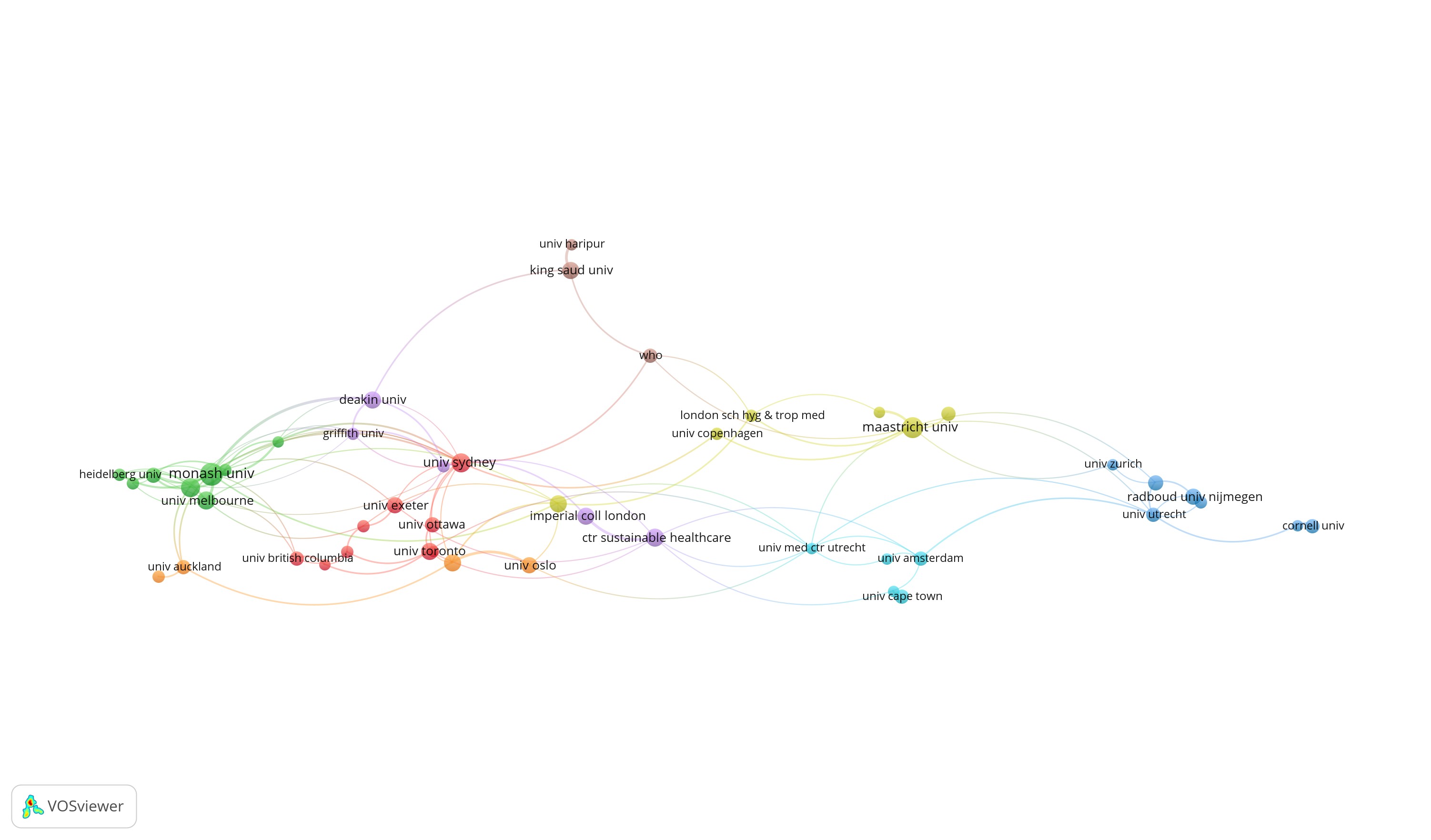

Supplement: Supplementary file 15 [file mmc15.zip › Supplemental Figure 4.jpeg]

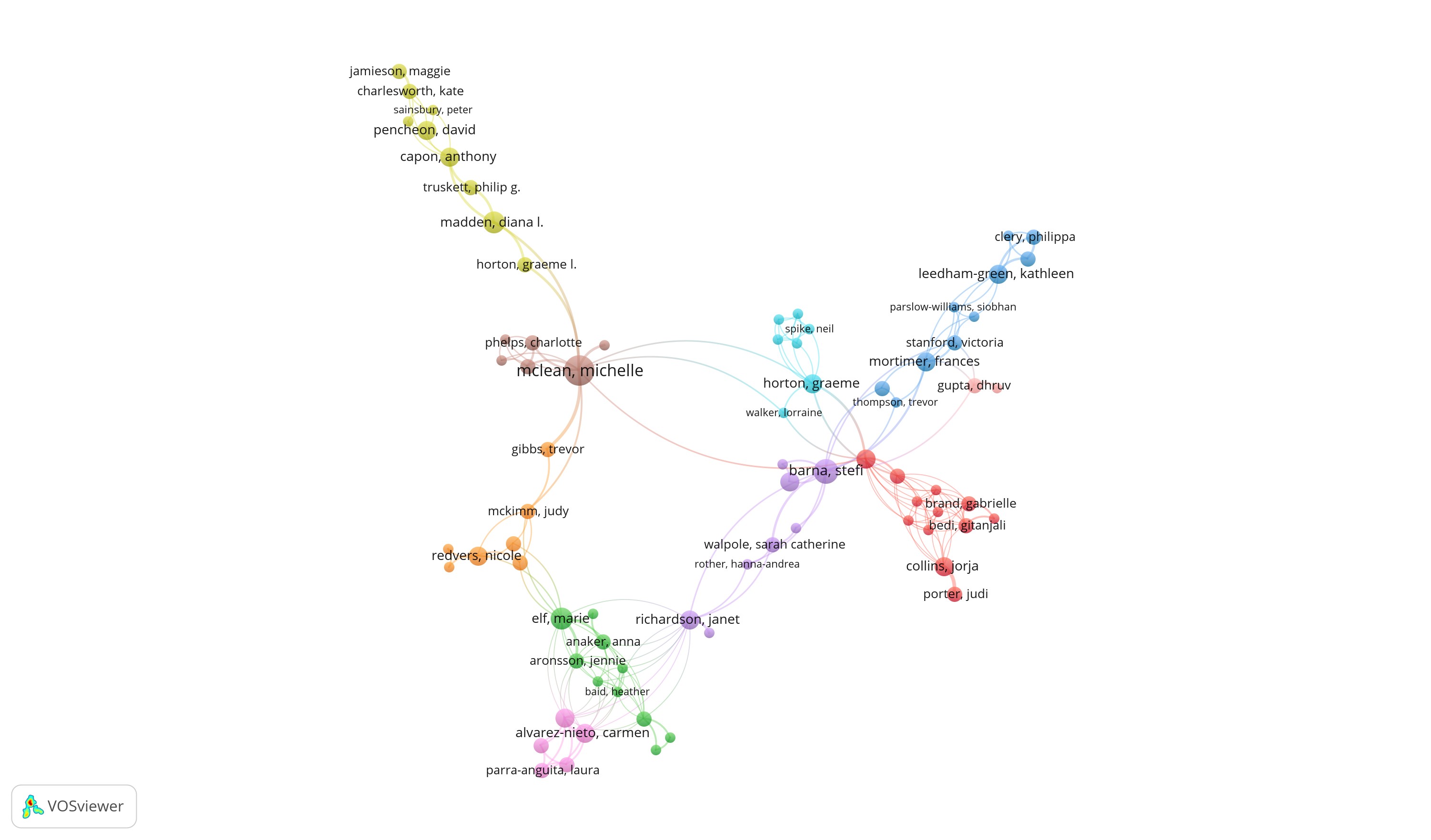

Supplement: Supplementary file 16 [file mmc16.zip › mmc16.jpeg]
